# Reframing pain: the power of individual and societal factors to enhance pain treatment

**DOI:** 10.1097/PR9.0000000000001161

**Published:** 2024-04-19

**Authors:** Rebeccah Slater, Christopher Eccleston, Amanda Williams, Katy Vincent, Mattias Linde, Michael Hurley, William Laughey

**Affiliations:** a Department of Paediatrics, University of Oxford, Oxford, United Kingdom; b Centre for Pain Research, University of Bath, Bath, United Kingdom; c Research Department of Clinical, Educational & Health Psychology, University College London, London, United Kingdom; d Nuffield Department of Women's and Reproductive Health, University of Oxford, Oxford, United Kingdom; e Norwegian Centre for Headache Research (NorHEAD), Trondheim, Norway; f Regional Migraine Unit, Sahlgrenska University Hospital, Gothenburg, Sweden; g Population Health Research Institute, St George's University of London, London, United Kingdom; h Health Professions Education Unit, Hull York Medical School, University of York, York, United Kingdom; i Reckitt Benckiser Healthcare Ltd, Dansom Lane South, Kingston Upon Hull, United Kingdom

## Abstract

The effectiveness of analgesics can be increased if synergistic behavioural, psychological, and pharmacological interventions are provided within a supportive environment.

## 1. Introduction

Pain is a common experience in most people's lives, with few exceptions. However, most pain, the “everyday pains” of life, are not always symptomatic of a pathology or underlying condition. Although common, these everyday pains are not trivial and can significantly reduce quality of life. Examples of “everyday pains” include headache, musculoskeletal pain (particularly back and joint pain), and pains associated with menstruation (alongside other female-specific pains). Assessment and effective management of these pains are important, not least because pain is the main reason people give for seeking formal health care.^30,32^

Pain is a complex, multidimensional experience; it is subjective, but associated behaviours are often visible to others. It goes beyond nociception to incorporate components of negative emotions and is highly variable across individuals, contextual settings, and societal structures. As such, it can be modified by a wide variety of pharmacological, physical, psychological, and contextual interventions. Healthcare providers, family, friends, and broader society have a role in shaping (framing) people's experience of pain and can influence the efficacy of pain-relieving interventions. These factors can also generate inequities in pain experience and treatment. Here, we discuss key approaches to enhancing pain-relieving treatments and, in contrast, discuss how societal biases can lead to suboptimal pain treatment. This perspective was inspired through discussion by a multidisciplinary group with expertise spanning psychology, paediatrics, women's health, neurology, and physiotherapy, which came together to discuss how pain can be better treated through accurate information provision, better recognition of pain, and by targeting the benefits of positive social interactions.

## 2. Harnessing the power of psychological and social factors

Pain and its interference are both shaped by psychological and social factors, derived from a wide range of individual, family, and societal influences.^32^ The subjective experience of pain is profoundly affected by our psychological framing, which is in turn influenced by our knowledge and beliefs, and the context and environment in which we experience pain. In randomised controlled trials investigating the efficacy of analgesic medications, placebo arms are used to control for the effects of expectations on treatment outcomes. However, more recently, we have begun to consider the powerful influence of expectations on real treatment outcomes. Remarkably, it has been estimated that up to 50% of the short-term treatment response to analgesics can be attributed to expectation rather than to pharmacodynamic effects.^5^ Positive expectations can substantially increase the effect of potent analgesics such as opioids. In the context of postoperative pain, priming of patients for a beneficial effect can significantly increase the effect of morphine,^14^ reduce the administration of patient-controlled analgesia,^36^ and double the effect of remifentanil in an experimentally induced pain paradigm.^6^ Positive expectations enhance nociceptive processing through activation of the descending pain modulatory system^20^ and through functional cortical brain networks. For example, disrupting the functional activity in the prefrontal cortex, using experimental techniques, can block expectation-induced analgesia^26^ and in Alzheimer disease, expectation-induced analgesia is less effective when there is reduced connectivity between prefrontal regions and other brain regions.^4^

Conversely, negative expectations can render a non-noxious stimulus painful^3^ and block the analgesic effect of opioids.^6^ Moreover, unrealistic positive expectations can be harmful, reducing the analgesic effect of interventions and damaging the fundamental relationship between the patient and care provider.^15^ Thus, accurately framing realistic individualised positive treatment expectations, through the provision of appropriate and targeted information, is an important goal.^27^ This is particularly important in marginalised groups, such as children, where dismissive comments such as *“it will not hurt”* during painful events can undermine coping and destroy trust.^42^ Congruence between treatment expectations of the person seeking pain relief and the care provider not only increases overall satisfaction^11,43^ but also can directly increase analgesic treatment effects. Social modulation of pain is possible through supportive and empathetic interactions, which is particularly feasible in the over-the-counter pharmacy setting, where care providers who interact with people experiencing pain have the opportunity to provide supportive and accurate advice. A recent neuroimaging study revealed that positive social interactions can engage descending pain modulatory brain regions, which reduces pain and is further heightened by greater therapeutic alliance.^20^ Fortunately, people's expectations—including of those experiencing pain—are highly dynamic and therefore modifiable through the provision of accurate information and supportive social frameworks.

## 3. Maximising the synergy of nonpharmacological and pharmacological interventions

The goal of reducing pain in others can be achieved by optimising the provision of high-quality verbal and written information regarding treatment properties, mechanisms, and effects; by ensuring empathetic communication with people experiencing pain; and by providing opportunities for observational learning, such as witnessing treatment success in others.^5,13^ Optimising expectations should be viewed as a therapeutic target that has the power to enhance analgesic outcomes, ultimately altering the long-term trajectories of pain conditions. However, there is a widely held belief within health care and wider society that pharmacological treatments are superior to nonpharmacological interventions, which may not necessarily be true; in reality, they are likely to work in synergy, so there is no need to choose one or the other. This is perhaps further compounded by the common use of the term “nonpharmacological,” defining a class of treatments by what they are not, rather than describing them in more specific terms such as psychological or behavioural interventions. By implication, pharmacological analgesia is upheld as the index modality by which all others are defined. Behavioural and psychological interventions can be highly effective in managing pain and its impact and can have a transformative effect on patient wellbeing and function.^9^ For example, in the management of migraine, there is now very good evidence for a range of psychological and behavioural interventions, including biofeedback, relaxation training, and cognitive behavioural therapy (CBT),^41^ which reduce the frequency and/or severity of attacks. These interventions are often preferred by patients^2^ and are now endorsed by the World Health Organization and recognised in EU guidelines.^40^ In joint pain, there is evidence to support the value of structured physical activity to reduce pain,^23^ but a dominant fear of further pain or damage often limits participation; combining education, social support, and pharmacological analgesics can together reduce this barrier enough to make a real difference to patient outcomes.

Across the spectrum of pain conditions, there is great potential for synergistic effects of behavioural, psychological, and pharmacological interventions. Therefore, designing treatment plans to holistically address the biopsychosocial factors contributing to the pain experience could maximise treatment outcomes. This can be practically demonstrated in an example protocol of interventions that can be used to treat pain caused by migraine, presented in Table 1, and a case study that makes use of this protocol, presented in Table 2.

**Table 1:** An example of a treatment protocol for migraines.

Recommendation	Basis
Educate patients about migraine, and where appropriate reassure	Many patients with migraine fear an underlying serious cause for their symptoms, which impairs the positive psychological framing of pain. All patients should receive an explanation of the nature of migraine, the goals of management, and where appropriate full reassurance of the nonsinister basis of migraine should be provided.^40^
Identify triggers for attacks	Migraine diaries can help patients identify potential triggers for attacks. However, there is need for balance, and the importance of triggers should not be overemphasised as they can become an unnecessary source of anxiety.^40^
Discuss aspects of measures to deal with stress, including relaxation	Stress can worsen the frequency and impact of migraine attacks. It can also impact sleep. There is evidence to support relaxation techniques, such as those based around breathing techniques, meditation, mindfulness, and biofeedback.^40^ There are now mobile apps available to perform biofeedback techniques at home, which may be more practical and less costly than visiting a healthcare professional, especially if multiple sessions are needed. There is also evidence that good nutrition, which supports the health of the microbiome, can benefit anxiety and mental health.^1^
Give nutrition advice	Patients have often heard that specific foods can trigger attacks. If they have seen clear links, it makes sense to avoid these foods. Food diaries may help. However, the relationship between migraine, food, and other triggers is complex, and the elimination of multiple foods is not always productive and can lead to nutritional deficiencies.^21^ There is some evidence for ketogenic, modified Atkin's and Mediterranean diets in migraine prevention, but more research is needed.^21^ The microbiome, gut brain axis, and probiotics are emerging areas of research interest. Evidence in migraine needs strengthening,^21^ but there are potential benefits for general health.^1^ Nutrition advice should reflect the general principles of healthy eating.
Recommend regular exercise	Although limited, there are some data that support aerobic exercise for the prevention of migraine.^21,40^ Exercise is also beneficial for general health. Acknowledge that some patients find rigorous exercise triggers an attack,^44^ and effort and duration may need to be adjusted accordingly. Research supports advice that patients work up to exercise at a moderate to high intensity level, 3 times a week for 30 min each session, excluding warm up and cool down.^45^
Discuss the use of cold packs during attacks	Some patients find the application of local cold to the forehead or temple helps relieve pain during the attack. This can also be achieved by applying cooling topical gels.^40^

**Table 2:** An example case study of migraine treatment.

A 30-year-old woman consults her General Practitioner with a 7-year history of migraine without aura. The attacks average 2 per month, lasting one or 2 days. Symptoms are severe enough to cause absence from work. Current acute treatments only partially relieve symptoms.
The doctor takes a medical history and conducts a focussed examination. She establishes the patient is concerned about a sinister cause for the pain. She reassures, based on the absence of clinical red flags, positively reframing the pain as a symptom of migraine, a common condition which is not dangerous but does require appropriate management. This is a significant relief to the patient. The diagnosis provides validation for the requirement for sickness absence days but also relief from the worry of serious illness.
In checking general health, the doctor ascertains that the patient is unhappy with recent weight gain, experiences work-related stress (exacerbated by the need for migraine absence days), and rarely exercises. In a supportive way, she explains to the patient that stress can exacerbate the frequency and impact of migraine attacks. She offers to provide the patient with a letter that can be given to occupational health. She also discusses relaxation techniques, including agreeing with the patient's suggestion of trying a mindfulness app. She explains there are some data that support regular exercise in migraine prevention and much more data that back the benefits of exercise for physical and mental health. As well as recommending exercise, she also provides healthy nutrition advice, with the smiling comment “this can only be good for your health, and it may help your migraines.”
Her advice on a new pharmacological treatment is delivered with equal positively, being honest about the medication, but avoiding any phrases that hamper the prescription with a nocebo message.
The patient leaves feeling reassured, validated, and relieved that the doctor is helping her situation at work. She is determined to adopt the healthy lifestyle measures that may also help her migraines. She also has a new pharmaceutical treatment to try. This treatment is important, but it is only one of the important things the doctor has done to manage the pain.

## 4. Promoting patient-relevant end points and “functional” improvements

For many pain conditions, achieving a pain-free state is often an unachievable expectation, the pursuit of which can be damaging. Focussing on reducing the impact of pain is often more realistic.^18^ Setting realistic and relevant treatment outcomes is important both in clinical trials of analgesic interventions and in clinical practice. The pain experience or analgesic effect is all too often reduced to a single category or number. This fails to account for the profound impact of pain on physical, emotional, and social functioning and on overall quality of life of the individual. A small reduction in pain may be sufficient to give a significant increase in function and therefore quality of life. Classically, patient-reported outcome measures (PROMs) have been most used in oncology research, capturing the effects of treatments on function and quality of life in the absence of a cure,^12^ and is well advanced in rheumatology.^33^ Given the parallels with chronic or recurring pain, over the past 20 years, there has been a drive towards the development of patient-reported outcomes (PROs) and PROMs. These outcomes need to be specific to the population to which they are applied, as constructs do not necessarily translate across conditions or populations.^10^ Thus, core outcome sets of PROs and PROMs have been and are being developed for a variety of pain conditions, facilitating comparison of effectiveness across trials and reducing outcome reporting bias.^37^ However, for these measures to effectively capture what people value most, they must be developed in direct consultation with people who suffer from pain,^39^ increasing their validity.^17^ Application of these measures ensures that clinical trials have the potential to identify interventions which truly benefit the target population, without unacceptable risks or adverse effects. This means targeting “functional” outcome measures towards the needs of different groups; eg, perhaps people with muscle or joint pain would benefit most from an intervention that increases their mobility—which could potentially be tracked using various outcome measures. An emphasis on “functional improvement” rather than an overreliance on numerical pain scales, where the complexity of pain experience is reduced to a single score, is a clear goal. This goal should be considered in the earliest stages of experimental design right through to regulatory decision making, where appropriate outcome measures are used to assert benefits of analgesic interventions. We note that wearable technology has great potential to monitor aspects, such as activity/mobility levels, and may add a valuable component to PROMs. For a recent paediatric pain example, see work by Palermo et al.^34,35^

## 5. Addressing pain-related expectations and biases in society

An individual's experience of pain is greatly influenced by the attitudes and behaviours of friends, family, healthcare professionals, local community, and wider society. Society is evolutionarily primed to be suspicious of “social cheating” where people do not pull their weight, but request help. This suspicion can result in gross underestimation or dismissal of pain by friends and family and in health care and stigmatisation when people express their pain to others.^24^ Suspicion is compounded by the invisibility of pain and reliance on self-report. Most members of society understand pain through their own acute pain experiences in which the symptom arises from tissue damage and resolves with healing. However, ongoing pain deviates from the classic biomedical model of illness and violates society's expectations.

People suffering from ongoing pain are often forced to justify their pain when seeking access to health care, and widely held ideals of stoicism, acceptance of suffering, and fighting pain can dramatically hinder the honest expression of pain and result in its undertreatment. Conversely, if people overtly display their pain to convey a sense of suffering, this can be misinterpreted as an exaggeration of their grievance, or worse, of malingering. Furthermore, the fundamental bias towards the minimisation of others people's pain is intensified in certain groups of society, and this has led to well-documented inequity in pain recognition and pain management according to age,^19^ sex,^25,38^ disability,^16^ ethnicity,^29^ and culture,^31^ all contributing to inequity in health care. For example, female-specific pain conditions are underresearched, stigmatised, shrouded in secrecy and embarrassment, and often dismissed as “a normal part of being a woman,” failing to acknowledge the substantial impact of those pain conditions on the individual and on broader society. In paediatrics, despite reports of greater pain intensity in children with cognitive disabilities, pain is assessed less frequently and treated with fewer opioids in these children.^7,28^

Addressing society's deeply entrenched biases and the stigmatisation of pain will require a significant effort both within health care and in communities. An unwritten “societal contract” temporarily excuses an individual with pain from societal responsibilities, but this is coupled with an expectation of efforts to recover.^24^ Observed behaviours that fall outside this expectation can be frowned upon, even if they are important steps towards an individual's recovery—eg, adversely judging a person on sickness leave with back pain for taking a walk in the park. We have an opportunity to substantially reduce pain in others by increasing awareness and understanding of pain, providing better education and training about optimal pain communication, and by encouraging compassionate care.^8,22^

## 6. Conclusion

Pain following acute injury is protective; it can prevent us from further damaging injured limbs, can educate us, and can provide a signal to others to encourage their care and protection. However, when pain is experienced long after or without injury, it is more difficult to articulate, and normal social interactions and behavioural expectations can be distorted. Pain will likely affect everyone at some point in their lives, and taking important steps to recognise how we can best respond to people who are experiencing pain can help alleviate suffering in others. This includes adopting compassionate and supportive approaches to people in pain, providing high quality and accurate information tailored to specific pain occasions (which includes advice on cognitive and behavioural interventions) and identifying relevant, realistic functional improvements by way of demonstrating meaningful relief. These interventions can be combined with pharmacological approaches to pain treatment to deliver synergistic benefits, and both should be considered as part of the overall suite of interventions.

## Disclosures

W.L. is employed by the Reckitt group of companies, which owns and distributes the Nurofen and Biofreeze brands. This paper is based on the outputs of 3 virtual meetings which formed an advisory board on aspects of pain and analgesia which go beyond pharmacology. All authors were participants in the advisory board, which was funded by the Reckitt group of companies, which owns and distributes the Nurofen and Biofreeze brands.
